# Iliac Stemmed Cups: A Review of History, Indications, and Clinical Outcomes in Revision Hip Arthroplasty and Primary Severe Dysplasia

**DOI:** 10.3390/jcm14144955

**Published:** 2025-07-13

**Authors:** Pier Giorgio Vasina, Paolo Palumbi, Ideal Frakulli, Christos Christoforidis, Claudio D’Agostino, Alberto Di Martino, Cesare Faldini

**Affiliations:** 1 Casa di Cura Malatesta Novello, 47521 Cesena, Italy; piergiorgiovasina@gmail.com (P.G.V.); paolo.palumbi@alice.it (P.P.); ifrakulli@yahoo.it (I.F.); 2 Orthopedic Clinic Thriasio General Hospital, 19600 Athen, Greece; christoforidismd@gmail.com; 3 1st Orthopedic Department, IRCCS—Istituto Ortopedico Rizzoli, 40136 Bologna, Italy; claudio.dagostino@ior.it (C.D.); cesare.faldini@ior.it (C.F.); 4 Department of Biomedical and Neurimotor Sciences, University of Bologna, 40126 Bologna, Italy

**Keywords:** Iliac stemmed cup, revision THA, revision hip arthroplasty, pelvic reconstruction, acetabular bone loss, developmental hip dysplasia

## Abstract

**Background:** The increasing incidence of revision total hip arthroplasties (rTHAs), particularly due to failure of the acetabular components and severe bone loss, necessitates reliable surgical solutions. Iliac stemmed cups (ISCs) have emerged as effective options for managing complex pelvic defects, including Paprosky type 3A and 3B acetabular defects, severe developmental dysplasia, and selected pelvic discontinuities. This review examines the historical evolution, clinical indications, and outcomes associated with ISCs. **Methods:** This narrative review analyzed the historical and recent literature concerning various ISC designs. We critically assessed clinical outcomes, complication rates, and implant survival from 13 key studies. **Results:** ISCs have progressed significantly from initial monobloc designs to contemporary modular configurations, substantially enhancing surgical versatility and biomechanical stability. Clinical outcomes varied with reported complications such as infection, dislocation, mechanical failure, and aseptic loosening ranging from 10% to over 30%. Newer modular implants like the Sansone cup have demonstrated improved outcomes, with complication rates below 10% and five-year survival rates exceeding 95%. **Conclusions:** ISCs are reliable and versatile implants, particularly suited to address significant pelvic bone deficiencies. Optimal surgical techniques and careful implant selection remain essential to minimize complications and achieve favorable long-term functional outcomes, making these implants valuable tools in complex hip arthroplasty.

## 1. Introduction

The number of primary total hip arthroplasties (THAs) has increased steadily over the last few decades. Consequently, the number of revision total hip arthroplasties (rTHAs) is expected to grow considerably in the coming years [1,2]. rTHA is a demanding surgery that requires the orthopedic surgeon to have significant experience and specialized reconstructive skills to optimize outcomes and overcome the difficulties arising from the wide variability and potential severity of the anatomopathological presentation, the technical challenges of the surgical process, and the elevated rates of complications [3].

More than one-third of rTHAs are due to the failure of the acetabular component [4,5]. The main causes leading to revisions include aseptic loosening, dislocation, septic loosening, and metallosis, usually with the presence of pseudotumor and migration of the acetabular component, among others [6]. Most failures are associated with varying degrees of bone loss. This bone loss can be secondary to a preexisting bone deficit before primary implants, as seen in dysplastic hips or cases of infection, aseptic loosening, and acute post-traumatic THA performance. Alternatively, bone can be lost during the removal of the acetabular component, creating a bone void that can hinder the surgeon’s ability to reconstruct acetabular anatomy and achieve primary cup stability [7,8,9]. The usual fixation sites may be missing or severely damaged, making the procedure technically challenging and potentially compromising its results.

In revision surgery of the acetabular cup, the primary goals are to ensure the stability of the acetabular component and restore the center of rotation of the hip joint. Achieving these goals helps prevent implant dislocation, aseptic loosening, and migration. The precise and careful planning of the operative procedure is essential. Among the several steps required to perform rTHA surgery, analyzing and classifying any bone defect pre- and intraoperatively is crucial for accurate acetabular reconstruction [10].

Various techniques have been proposed for managing large defects, including the placement of an uncemented cup at a high hip center [11], jumbo cups [12,13,14,15], anti-protrusion cages [16,17,18,19], highly porous metal cups [20,21,22,23,24,25], oblong cups [26,27,28,29,30,31], custom triflange cups [32,33,34], cup–cage constructs [35,36,37], and stemmed cups [38,39,40,41,42,43,44,45,46,47,48].

ISCs have been used for many years in tumor resection cases [49], and numerous designs have been developed. These include the Coned Hemi-Pelvis, also called “Ice Cream Cone” (Stanmore Implants, Elstree, UK) [50,51], the McMinn cup (Link, Newsplint, Basingstoke, UK) [52], the Ring prosthesis (Zimmer, Swindon, UK) [38,39], the titanium pedestal cup (Zimmer, Warsaw, IN, USA) [53], the modular reconstructive cup (ModuRec system, Zimmer, Warsaw, IN, USA) [48], the Integra cup with peg (Lépine, Genay, France) [48], the LUMIC pedestal cup (Implantcast, Buxtehude, Germany) [54], and others.

All these designs are based on the principle that an iliac stem attached to the component helps reduce shear forces that may compromise stability, while also restoring the anatomical continuity between the pelvis and lower limb by anchoring into the iliac isthmus (also referred to as the ilio-pubic bar). This structure is defined as the thickened portion of the ilium located between the acetabular roof and the sacroiliac joint [47]. Testut and Latarjet [55] described it as a long bone with a short diaphysis and a medullary canal. Other studies have demonstrated that the iliac isthmus remains intact even in cases of severe bone loss. By implanting the stem into the intramedullary space of the isthmus, this design facilitates the restoration of both anatomical and biomechanical continuity between the spine and the lower limb in cases of extensive acetabular defects [47,56].

**Figure 1:**
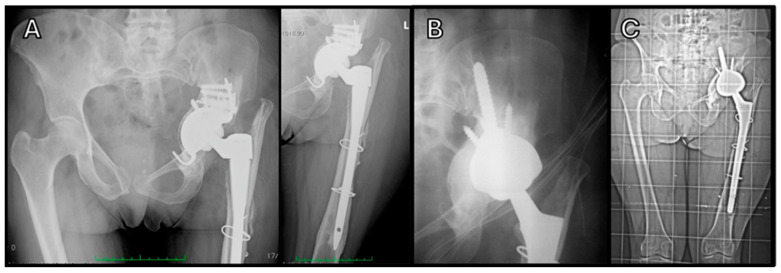
Revision of acetabular cup and femoral stem: (**A**) Preoperative image showing acetabular cup mobilization and femoral stem subsidence. (**B**) Postoperative radiograph illustrating the use of an iliac cup, cancellous bone graft, and Wagner femoral stem via a posterior surgical approach, demonstrating complete bone graft integration at six months. (**C**) Comparative images of preoperative status and postoperative outcome at one-year FU.

The iliac stem, positioned along the physiological lines of load, achieves good primary stability by transforming most of the tangential stresses into normal contact forces [57]. The restoration of the center of rotation may be achieved in combination with the impaction bone grafting technique. If correctly pressurized, grafts are not overloaded and reabsorbed; instead, these integrate with the bone, recreating a good bone stock even in cases of severe aseptic loosening (Figure 1).

The aim of this narrative review is to describe the historical development of ISC designs, providing a broader perspective on the use of these implants in revision hip arthroplasty. In particular, it aims to highlight their use not only in oncologic cases—which represented the most common indication—but also in other complex conditions associated with bone defects, such as severe hip dysplasia.

## 2. Indications

**Figure 2:**
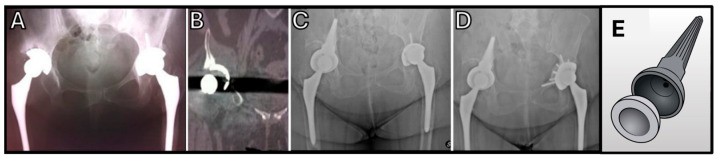
Bilateral revision for polyethylene (PE) cup failure due to gamma-ray sterilization: (**A**) Radiograph demonstrating bilateral acetabular cup mobilization. (**B**) Computed tomography (CT) scan illustrating polyethylene protrusion into the pelvis on the right side. (**C**) Radiographic view post revision with a pedestal cup implant via a posterior approach. (**D**) One-year postoperative follow-up after left side revision. (**E**) Schematic illustration of the pedestal cup design by Perka and Schoellner.

The aim of using ISCs is to restore the hip rotation center in cases of significant pelvic defects, avoiding the use of structural bone grafts. According to the Paprosky classification, the main indications for ISCs are type 3A and 3B pelvic defects and, in some cases, dysplastic acetabulum type 2B and 2C. Its application is also possible in patients with pelvic discontinuity in which the posterior column and iliac crest are still preserved (Figure 2).

## 3. History

**Figure 3:**
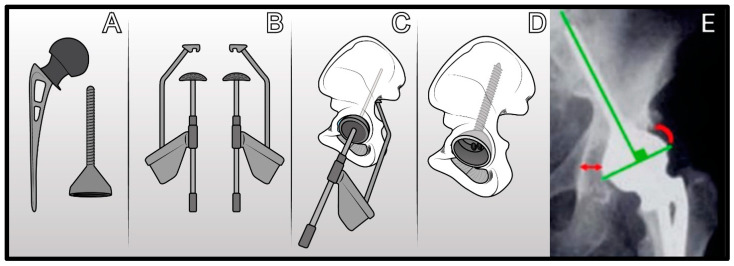
First-generation Ring cup prosthesis: (**A**) Uncemented metal-on-metal Moore prosthesis (Ø 40 mm) with screw fixation. (**B**,**C**) Drill guides utilized for implantation. (**D**) Intraoperative lateral view demonstrating prosthesis positioning. (**E**) Radiographic evidence illustrating lateralization of the cup placement.

Acetabular cups with iliac fixation were introduced in the late 1960s by Ring [38]. This first-generation cup featured a metal-on-metal, uncemented, screwed, monoblock design. It was used interchangeably for both the right and left hip and was coupled with the standard 40 mm Moore prosthesis. In a series of 155 cases with a maximum follow-up of 4 years, Ring reported only one mechanical failure. Positive results were confirmed by Patterson [58], who reported an 8.7% revision rate in a series of metal-on-metal hips with follow-up ranging between 5 and 20 years. A critical issue was the lateralization of the center of rotation, as the same cup was used for both the right and left hip (Figure 3).

**Figure 4:**
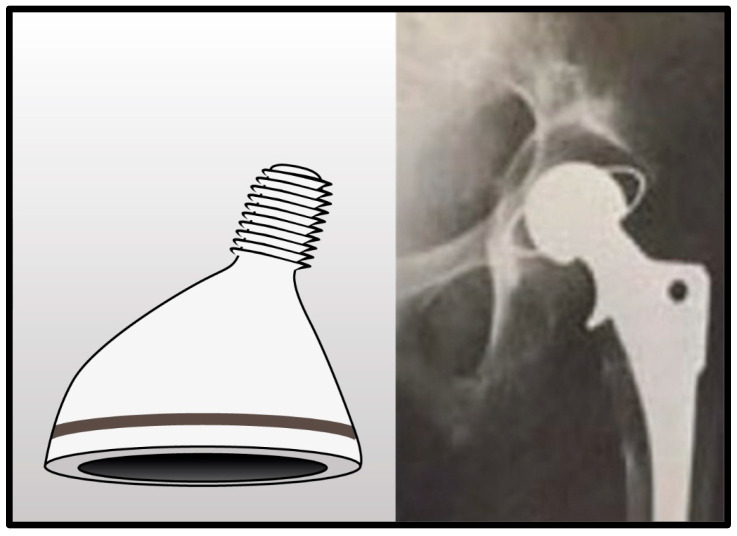
All-polyethylene acetabular component with a 32 mm femoral head and angled peg impacted into bone. Radiographic image demonstrates component failure associated with significant osteolysis.

In 1983, Ring et al. [39] presented a second-generation iliac cup designed to achieve primary stability at the iliac isthmus. These were characterized by an all-polyethylene (PE) cementless design that could be impacted into the bone with an angled peg and a 32 mm head [34]. Despite introducing right and left versions to avoid the problem of lateralization of the center of rotation, this uncemented cup was unsuccessful due to the development of osteolysis (Figure 4).

In the early 1990s, McMinn developed an evolution of the original Ring design, consisting of an uncemented titanium monobloc component coated with hydroxyapatite (HA) and PE inserts [59]. The stem had a very good press fit, allowing for good cancellous bone graft integration. A cemented version was also devised. Although McMinn et al. [52] initially reported excellent results, several technical flaws emerged, particularly the peg’s rounded shape and the lack of dual mobility and optional screws to improve stability. This led to numerous clinical failures and mixed results [41,42].

**Figure 5:**
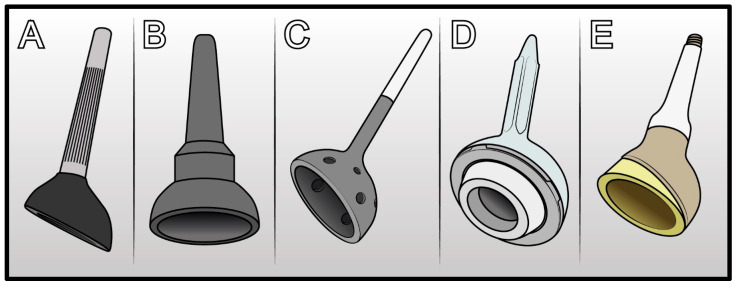
Evolution of cone-shaped acetabular cup: (**A**) McMinn cone prosthesis. (**B**) Schoellner cup cone prosthesis (Zimmer-Biomet). (**C**) Stanmore cone prosthesis. (**D**) Integra cone prosthesis (Lépine). (**E**) LUMiC cone prosthesis (Implantcast).

A new design, named the Pedestal cup, was introduced in 1999 by Perka and Schoellner [43,53]. It consisted of a titanium monobloc with a 45-degree inclination of the stem, which was also endowed with antirotating longitudinal ribs (Figure 5). Perka and Schoellner presented a series of 51 hip revisions, reporting a 23% complication rate. This design was later used in bone tumor cases [60]. In 2016, Stihsen et al. [61] used the Schoellner pedestal component (Zimmer, Freiburg, Germany) for the reconstruction of major acetabular defects in 35 patients. A total of twenty-six patients (74%) experienced aseptic loosening or another mechanical failure, four (12%) had an infection, and five (14%) underwent a periprosthetic fracture at an average follow-up of 63 months. In this retrospective study, the cumulative five-year survival for aseptic loosening was 94% in patients without pelvic discontinuity and 56% in those with pelvic discontinuity.

Similar iliac cups derived from the Pedestal cup were introduced over the years, including the Procotyl Z (Wright) and OMNIA (Adler Ortho) [62].

In 2003, the Stanmore cone prosthesis (Stanmore Implants, UK) was introduced, featuring a design similar to the McMinn component but with changes to the surface coating and the stem aimed at providing rotational stability and improved loading [61,62] (Figure 5C). Matharu et al. [50] described a modified surgical technique for the reconstruction of major acetabular defects in 28 patients (15 oncologic and 13 non-oncologic patients) using the Stanmore ‘ice-cream’ cone prosthesis and reported its short-term clinical outcomes. Postoperative complications occurred in 14% (*n* = 4). The main complications described with the use of this implant were infection and hip dislocation. The ISC provided a relatively safe method for acetabular reconstruction (including pelvic discontinuity) in patients requiring oncological reconstructions or hip revision arthroplasty.

In 2003, the Integra cup (Lépine, France) was launched. It was characterized by a higher inclination of the stem to the axis of the shell compared to previous designs [47]. The implant and peg were entirely coated with porous titanium and a layer of hydroxyapatite. The peg intended for the iliac isthmus had a star-shaped cross-section to augment rotational stability. The cup accepted a dual mobility polyethylene liner, and additional cancellous bone screws were used [63] (Figure 5D).

Issa et al. in 2020 [63] performed a retrospective study in which sixteen Integra TM cups were implanted in 14 patients with aseptic acetabular loosening combined with severe acetabular bone loss. At a mean follow-up of 48.8 ± 23.4 months, six hips experienced one or more complications (37.5%), including three infections (18.8%), two mechanical failures (12.5%), and one dislocation (6.7%). The cup had to be removed in three patients (18.8%). These complications required reoperation, resulting in a cumulative incidence of revision for any reason at 5 years of 31%. They concluded that despite the high complication and revision rates, the stemmed acetabular cup is a viable alternative in salvage reconstruction procedures.

**Figure 6:**
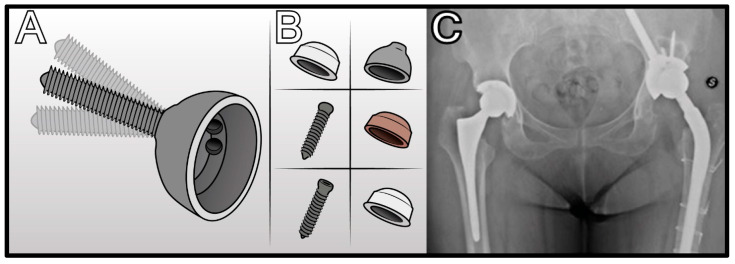
Sansone modular iliac cup system: (**A**) Demonstration of the wide range of rotational and angular adjustments enabled by the Morse taper connection between the cup and iliac screw. (**B**) Ceramic-on-ceramic bearing option with hydroxyapatite (HA)-coated iliac screw. (**C**) Clinical application of the ISC in a revision case performed via a posterior approach (first case treated in 2009).

More recently, the LUMiC (Implantcast, Germany) and the Sansone cup (Citieffe, Calderara di Reno, Italy) were developed, introducing modularity by separating the stem and acetabular cup to improve joint stability and versatility [54,64]. An innovative feature of the Sansone cup was a 50° polyaxial freedom between the iliac screw and the hemispherical cup [64]. The cup accepted either polyethylene or ceramic inserts (Figure 5E and Figure 6) and was equipped with screw holes for additional screw fixation to avoid rotation and promote primary stability, particularly during the surgical phase of locking the main iliac screw. In 2017, Cadossi et al. [64] reported favorable results for the Sansone cup in patients with large acetabular bone defects at 2 to 7 years follow-up (average follow-up of 46 months). They evaluated a series of 121 hips, reporting complications in 10 hips (7.5%), including aseptic loosening in 1 hip (0.8%), dislocation in 3 patients (2.5%), and 5 deep infections (4.2%). In five patients, the cup was removed, and the estimated survival rate at 5-year follow-up with implant removal for any reason was 95.6%.

**Figure 7:**
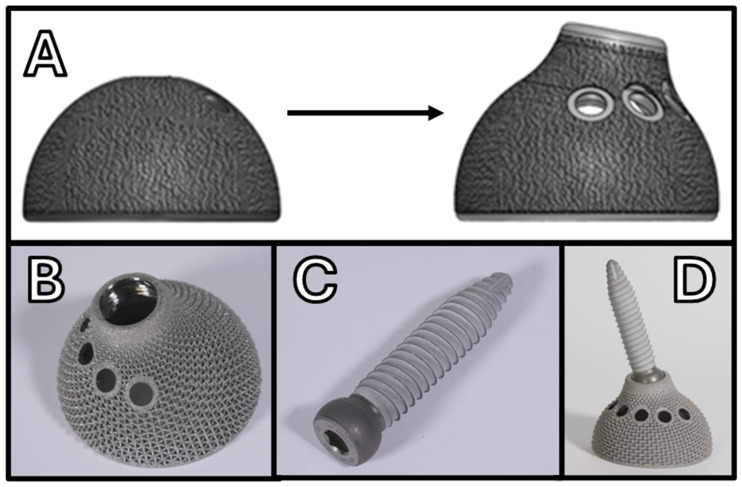
Evolution of the Medacta iliac screw cup system: (**A**) Comparison between the original hemispherical Mpact^®^ cup and the derived Iliac Screw cup. (**B**–**D**) Latest generation of the Iliac Screw Mpact^®^ 3D Metal design, showcasing advancements in modularity and the 3D-printed structure.

**Figure 8:**
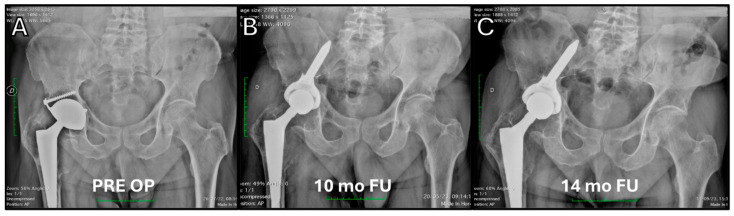
Acetabular cup revision for severe osteolysis in the periacetabular region and proximal femur. (**A**,**B**) The procedure was performed using the Iliac Screw Mpact^®^ 3D Metal cup and proximal femoral bone grafting via anterior approach. (**C**) Follow-up at 14 months demonstrates successful bone graft integration and stable cup fixation.

In 2020, Medacta introduced the Iliac screw Mpact^®^ 3D Metal, combining the modular big polar screw of the Sansone cup for primary fixation in the iliac portion of the pelvis with the external titanium alloy 3D metal structure, realized through 3D printing technology and derived from the existing Mpact 3D Metal acetabular cup (Figure 7). Among the most relevant features of this cup are its modularity and the availability in two versions, set at 10° and 25°. The latter, designed to treat patients with severe dysplasia, is also available in small sizes. These cups all have bone screw holes to help prevent rotation. Unlike other implants, a specific instrument allows the cup to be held while the locking main screw and washer are tightened. The company developed an ancillary instrumentation set, making it suitable for implanting the ISC through an anterior approach, reducing wound length and muscular damage (Figure 8).

## 4. Surgical Technique of Iliac Stemmed Cup

**Figure 9:**
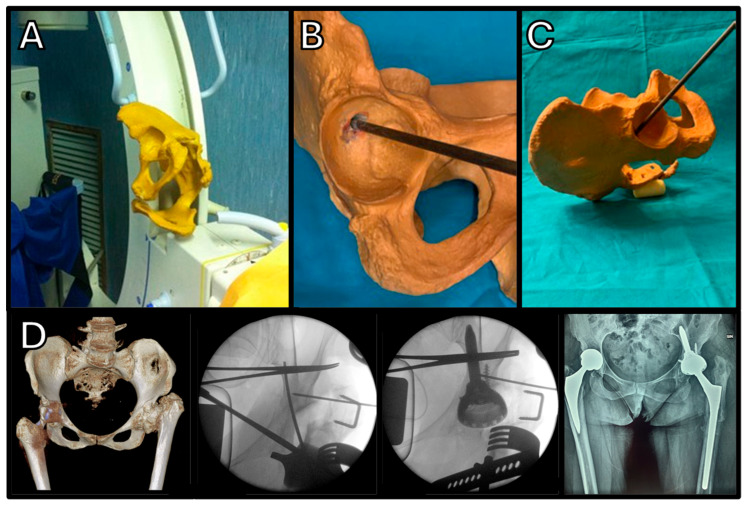
Surgical technique to implant the Iliac Screw Mpact^®^ 3D Metal cup in a patient with severe varus deformity of the femoral neck and a dysplastic acetabulum. (**A**–**C**) The model represents the patient’s position and shows the entry point for the iliac isthmus. (**D**) The procedure was carried out via a posterolateral approach, including a femoral shortening osteotomy to restore limb length.

Accurate preoperative planning with X-rays and CT scans is required before the implantation of ISC implants. To identify the iliac screw entry point, which lies approximately 1.5 cm anterior to the greater sciatic notch (Figure 9), a pilot hole is made with a starter awl, and the integrity of the iliac isthmus is carefully assessed with a thin metallic probe and by digital palpation around the sciatic notch. One of the key steps for successfully performing the procedure is the correct positioning and alignment of the stem within the ilium, as it is a major determinant of the primary stability of the cup. An intraoperative image intensifier is used to check the correct position of the probe. The progressive reaming of the iliac isthmus is performed using handheld reamers until cortical contact is achieved. The cup is implanted with the iliac stem at more than 50° of abduction. The iliac screw, inserted through the cup into the iliac isthmus, is fixed first by the main locking screw and then by the washer. The iliac screw diameter must be the same for Mpact^®^ 3D Metal or 2 mm more than the reamer’s size for Sansone.

The intraoperative anatomy may be complex and, as demonstrated in several studies [65,66,67], having a plastic pelvis model close to the surgical field can help surgeons visualize the position of the iliac bone and choose the correct stem alignment and positioning, especially in the first few cases. Identification of the entry point is easier if there is significant bone loss, compared to dysplastic cases, which typically display more severe deformities and fewer anatomical landmarks, as seen in difficult Eftekhar grades C and D, and in Crowe grades III and IV patients. The procedure can be performed using either an anterior or a posterolateral approach and can be coupled, if required, with a subtrochanteric shortening osteotomy, which may be necessary for the reconstruction of severely dislocated hips [68].

## 5. Use of Iliac Stemmed Cup in Severe Acetabular Dysplasia

Total hip arthroplasty for osteoarthritis secondary to severe developmental dysplasia of the hip (DDH) is a demanding procedure. According to the Eftekhar classification system (published in 1978), the severity of DDH is divided into four stages based on the degree of femoral head dislocation: type A features a slightly elongated acetabulum and flattened femoral head; type B and C represent intermediate and high dislocations, respectively, with poorly developed acetabulum; and type D describes an old, unreduced dislocation where the femoral head is not in contact with the ilium [69].

In 1979, Crowe et al. introduced another four-stage classification system for DDH, defining the degree of dislocation based on the percentage of the proximal displacement of the femoral head relative to pelvic height (I: <0.1%; II: 0.1–0.15%; III: 0.15–0.20%; IV: >0.20%) or the amount of subluxation (I: <50%; II: 50–75%; III: 75–100%; IV: >100%) [70].

The goal of THA in DDH is to lower and medialize the center of rotation of the hip, thereby restoring gluteal muscle function. The shallow, small socket characteristic of a severely dysplastic pelvis hampers the correct positioning and coverage of the femoral head when using standard acetabular cups. To address this, several surgical techniques involving bone graft augmentation are used to improve cup coverage and primary stability with standard cups [71,72], restoring the native center of rotation of the hip joint. Alternatively, the use of metal augmentations and custom implants can enhance the primary stability of the construct.

**Figure 10:**
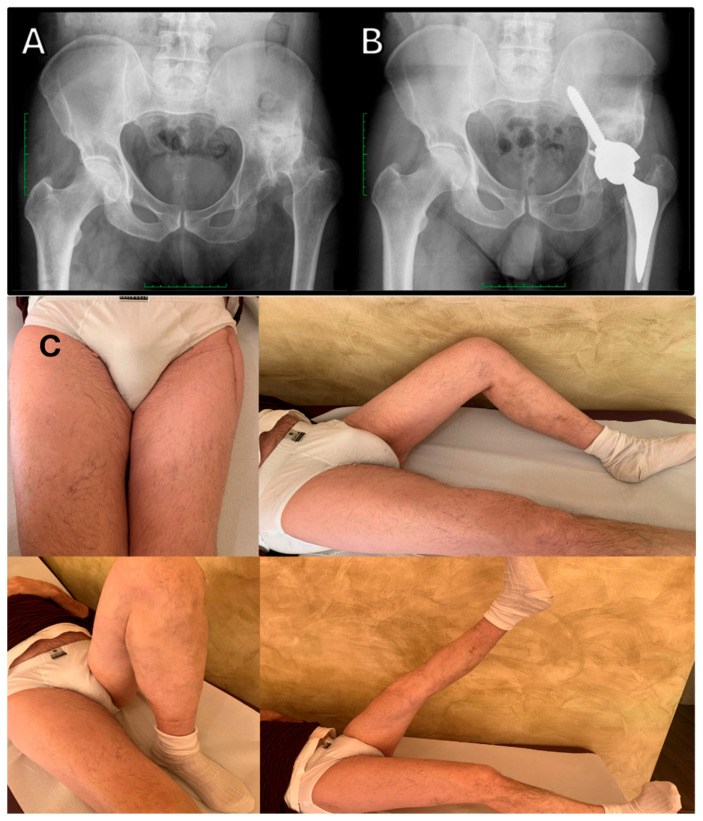
Restoration of hip biomechanics following iliac screw cup implantation: (**A**) Preoperative radiograph showing high hip center and dysplastic anatomy. (**B**) Postoperative anteroposterior X-ray at 2-month follow-up demonstrating medialization and lowering of the hip center of rotation, enabling normal ambulation without Trendelenburg lurch. (**C**) Clinical outcome at 1-month follow-up showing full recovery of hip range of motion.

In cases of incomplete cup coverage and dysplastic true acetabulum, iliac screw cups can help overcome these challenges by anchoring the acetabular cup to the iliac isthmus, thereby restoring the native center of rotation of the joint [64], allowing for immediate and complete weight bearing (Figure 10).

## 6. Outcomes

This narrative review also examines the recent literature on various ISCs designs. An electronic search was conducted in major healthcare databases, including PubMed, EMBASE, and SCOPUS, using the keywords “Iliac Stemmed Cup”, “Ice-Cream Cone Prosthesis”, “Stemmed Acetabular Cup”, “Pedestal Cup”, and “Iliac Stem”. The search targeted historical studies as well as prospective and retrospective articles published in English, with no date restrictions. The final search was completed in May 2025 and yielded 418 results.

After screening titles and abstracts, the authors critically selected 13 studies that reported outcomes of ISCs in the reconstruction of acetabular defects. Studies that did not report comprehensive data were excluded; if essential data were missing, the entire study was not considered for inclusion.

Most articles reporting on the clinical application of ISCs focused on small patient series, also including both post-oncologic and non-oncologic bone defects (Table 1).

ISCs have progressed significantly from initial monobloc designs to contemporary modular configurations, substantially enhancing surgical versatility and biomechanical stability. Clinical outcomes varied with reported complications such as infection, dislocation, mechanical failure, and aseptic loosening ranging from 10% to over 30%.

For the same grades of bone defects, a comprehensive systematic review by Aprato et al. [74] assessed the outcomes of acetabular revision surgeries using various reinforcement devices, including the Burch–Schneider cage, Müller ring, and Ganz ring, revealing suboptimal results, with an overall implant survival rate of 80% at a mean follow-up of 8.2 years. These results align with findings from several other studies [75,76,77,78,79]. More recently, Malahias et al. [80] reported even less encouraging data regarding the performance of the modern Burch–Schneider anti-protrusio cage, noting a survival rate of 90.6% at short term (2–5 years), 85.6% at mid-term (5–10 years), and a notable decline to 62% beyond 10 years.

Alternative strategies for addressing acetabular defects include the use of tantalum metal augments and trabecular titanium shells. These have shown favorable outcomes in cases of moderate bone loss [81,82]; however, in scenarios involving extensive acetabular destruction, long-term survivorship drops significantly, with one study reporting a 10-year survival rate of only 61.6% [83].

Comparing the strategies, newer modular implants like the Sansone cup have demonstrated improved outcomes, with complication rates below 10% and five-year survival rates exceeding 95% [64].

## 7. Conclusions

ISCs have shown satisfactory functional outcomes and represent a valuable option for orthopedic surgeons. One of their key advantages lies in the use of preserved bone stock from the iliolumbar bar and posterior ilium, even in cases of advanced bone loss. When performed by experienced surgeons, ISCs offer a safe and adaptable solution for managing Paprosky 3A and 3B defects, complex dysplasia (Eftekhar C and D; Crowe III and IV), and certain fracture cases in elderly patients.

ISCs can be used in cases of pelvic discontinuity where the posterior column and iliac crest are still intact. Compared to massive structural grafts, they permit immediate partial weight bearing, enhance the restoration of the anatomical center of rotation, and reduce the need for femoral components revisions, minimizing mechanical complications and undesired strain on the neurovascular bundle and soft tissues.

Future developments should aim to strengthen the biomechanical rationale of ISCs through advanced finite element analyses and experimental validation [84]. The incorporation of 3D-printed anatomical models and patient-specific intraoperative guides holds [85] promise for improving surgical precision and customization. Additionally, high-quality analyses and long-term clinical studies are needed to further clarify indications, optimize implant design, and standardize surgical protocols, ultimately enhancing outcomes in complex acetabular reconstructions.

## Figures and Tables

**Table 1:** Summary of key clinical studies on iliac stemmed cups, detailing indications, patient cohorts, follow-up duration, and reported outcomes.

Author and Year	Indication	Patients or Hips	ISC	Follow-Up	Results
P.A. Ring 1968 [38]	Dysplasia	155 cases	Ring Cup (Zimmer)	4 Years	Only 1 failure.
M. Patterson 1987 [58]	Dysplasia	156 cases	Ring Cup (Zimmer)	5–20 Years	Revision rate of 8.7%.
P. Badhe and P.W. Howard 2000 [41]	Severe acetabular deficiency	29 cases	McMinn Cup	46 Months (14–74)	Encouraging restoration of bone stock. No aseptic loosening.
Schoellner et al. 2000 [53]	Acetabular defects; resection arthroplasty	51 hips	Pedestal cup	1–5 Years	Few implant-related complications (first-generation screw failure and mispositioning of the pedestal).
Eisler et al. 2001 [42]	Gustilo–Pasternak grades 2, 3, and 4	26 hips	McMinn Cup	3 Years (1–5 Years)	Of the patients, 4 (17%) cups rerevised for aseptic loosening in the first 3 years. Another 9 cups (45%) were radiographically loose. Overall mechanical failure rate of 43.8% at a 3-year follow-up.
Perka et al. 2002 [43]	rTHA for high hip dislocation with extensive acetabular bone defects	4 patients	Pedestal cup	28.5 Months	There were no complications. At the latest FU, an HHS of 85 points was achieved, and all components were radiologically stable.
Tohtz et al. 2007 [73]	High-grade acetabular defects (Paprosky II and III)	50 hips	Pedestal cup	26 Months	Intra-op implant-associated complications: *n* = 3 (6%). Post-operative complications: hip dislocations (*n* = 10, 20%); septic loosening (*n* = 2, 4%); aseptic loosening (*n* = 6, 12%); implant migration (*n* = 7, 14%). Risk factors for implant loosening: missing reconstruction behind the pedestal cup; lateralization of the CoR; absence of the craniolateral wall; osteoporosis.
Willemse et al. 2010 [45]	Acetabular or global rTHA with severe preoperative acetabular deficiency	24 patients	McMinn Cup	4 Years (1–8 years)	Postoperative complications: sepsis (*n* = 3, 13%); dislocation (*n* = 2, 8%); mechanical failure (*n* = 9, 38%—dislocations incl.); aseptic loosening (*n* = 4, 17%). The center of rotation was restored in 75% of the hips. Adequate alignment was seen in 71%.
Fisher et al. 2011 [51]	Endoprosthetic replacement of the pelvis following tumor removal	27 patients	Stanmore cone prosthesis	39 Months (18–80 Mos)	Postoperative complications: dislocation (*n* = 4, 15%); deep infection (*n* = 3, 11%); local recurrence (*n* = 2, 7.4%); explant for severe pain (*n* = 1, 3.7%).
G. Matharu et al. 2013 [50]	Severe acetabular deficiency (oncological and hip arthroplasty patients)	28 patients	Stanmore cone prosthesis	12.5 Months	Complications occurred in 14% (*n* = 4).
C. Stihsen et al. 2016 [61]	Major acetabular defects	35 patients	Schoellner Pedestal Component (Zimmer)	63 Months	Postoperative complications: 11% infections; 14% dislocations; 17% loosening; 34% implant removed.
M. Cadossi et al. 2017 [64]	Major acetabular defects	121 hips	Sansone Cup (Cittiefe)	46 Months	Postoperative complications: 4.2% infections; 2.5% dislocations; 0.8% loosening; 4.2% implant removed.
Issa et al. 2020 [63]	Severe acetabular deficiency	14 patients	Integra Cup (Lepine)	48.8 Months (±23.4 Mos)	Postoperative complications: 18.8% infections; 6.7% dislocations; 12.5% loosening; 18.8% implant removed.

## Data Availability

No new data were created or analyzed in this study.

## Abbreviations

The following abbreviations are used in this manuscript:

THA	Total Hip Arthroplasty
rTHA	Revision Total Hip Arthroplasty
ISC	Iliac Stemmed Cup
DDH	Developmental Dysplasia of the Hip
CoR	Center of Rotation
